# TriDTI: tri-modal representation learning with cross-modal alignment for drug–target interaction prediction

**DOI:** 10.1093/bib/bbag034

**Published:** 2026-02-05

**Authors:** Gwang-Hyeon Yun, Jong-Hoon Park, Young-Rae Cho

**Affiliations:** Department of Software, Yonsei University Mirae Campus, 1 Yeonsedae-gil, Wonju-si, Gangwon-do, 26493, Republic of Korea; Department of Software, Yonsei University Mirae Campus, 1 Yeonsedae-gil, Wonju-si, Gangwon-do, 26493, Republic of Korea; Department of Software, Yonsei University Mirae Campus, 1 Yeonsedae-gil, Wonju-si, Gangwon-do, 26493, Republic of Korea; Department of Digital Healthcare, Yonsei University Mirae Campus, 1 Yeonsedae-gil, Wonju-si, Gangwon-do, 26493, Republic of Korea

**Keywords:** drug–target interaction prediction, tri-modal representation learning, modality alignment

## Abstract

The rapid advancement of artificial intelligence has positioned drug–target interaction (DTI) prediction as a promising approach in drug screening and drug discovery. Recent research has attempted to use pharmacological multimodal information to increase prediction accuracy. However, existing approaches are limited in fully utilizing more than three modalities, primarily due to information loss during the modality integration process. To overcome this challenge, we propose TriDTI, a novel framework that incorporates three modalities for both drugs and proteins. Specifically, TriDTI integrates structural, sequential, and relational modalities from both entities. To mitigate information loss during integration, we employ projection and cross-modal contrastive learning for modality alignment. Furthermore, we design a fusion strategy that combines soft attention and cross-attention to effectively integrate multimodal representations. Extensive experiments on three benchmark datasets demonstrate that TriDTI consistently achieves superior performance to existing state-of-the-art approaches in DTI prediction. Moreover, TriDTI exhibits a robust generalization ability across three challenging cold-start scenarios, effectively predicting interactions involving novel drugs, targets, and bindings. These results highlight the potential of TriDTI as a robust and practical framework for facilitating drug discovery. The source codes and datasets are publicly accessible at https://github.com/knhc1234/TriDTI.

## Introduction

Predicting drug–target interactions (DTIs) is a fundamental challenge in drug screening and drug discovery [1, 2]. Traditional drug discovery pipelines are often constrained by high costs and long development cycles [3–5]. To overcome these limitations, diverse computational methods have been proposed, enabling both deeper analytical insights, and more efficient prediction in DTI studies [6, 7]. These approaches can be broadly categorized into ligand-based, docking-based, and chemogenomic methods [8].

Ligand-based methods exploit structural similarities between ligands to infer DTIs, while docking-based methods estimate binding affinity by simulating the interactions between drug molecules and the 3D conformations of target proteins [9]. However, both methods are inherently restricted by the scarcity of experimentally verified ligands and reliable 3D structural data [10–12]. In contrast, chemogenomic methods address these limitations by directly leveraging molecular representations of drugs (e.g. SMILES) and protein sequences, thereby eliminating the reliance on 3D structural data or extensive ligand libraries. By enabling predictions for uncharacterized targets, this strategy greatly expands the applicability of computational drug discovery. Building on this foundation, deep learning models have emerged, offering diverse solutions for modeling DTIs. These models are commonly categorized by their treatment of drug embeddings into sequence- and structure-based methods [13].

Sequence-based methods predict DTIs directly from raw sequence data, typically encoding a drug’s SMILES code and a protein’s amino acid sequence into vector representations. For example, TransformerCPI [14] employs a Transformer architecture to jointly encode SMILES and protein sequences, generating predictions through a fully connected layer. HyperAttentionDTI [15] constructs feature matrices from each sequence using a convolutional neural network (CNN) block and captures complex noncovalent interactions between atoms and amino acids through an attention mechanism. More recently, DLM-DTI [16] leverages pretrained language models, specifically ChemBERTa [17] and ProtBERT [18], combined with a lightweight teacher–student learning strategy to enhance prediction efficiency. DrugKANs [19] proposed a novel paradigm that integrates Kolmogorov–Arnold Networks with sequential representations, demonstrating improved expressiveness, and interpretability in modeling complex drug–target relationships.

In contrast, structure-based methods represent drugs as molecular graphs, capturing structural information that sequence-based embeddings may overlook. For instance, MGraphDTA [20] utilizes a multiscale graph neural network (GNN) for molecular graphs alongside a multiscale CNN for protein structural features. Similarly, MGMA-DTI [21] applies a 2-layer graph convolutional network (GCN) to molecular graphs and a multi-order gated convolution to protein sequences, integrating these features through an attention-based fusion module. Furthermore, GPS-DTI [22] uniquely enhances drug representation by employing a GPS layer [23], though it relies on ESM2 sequence embeddings refined by CNNs for protein feature extraction. However, DTI involves complex interactions situated within a wider biological context, leading some studies [24–26] to explore leveraging graph representation learning over heterogeneous biological information networks to capture global dependency patterns. Despite these attempts to utilize relational information, existing sequence-based and structure-based methods primarily rely on single-representation paradigms. Although computationally efficient due to their reliance on a single representation, these approaches are limited in capturing the full spectrum of multimodal information inherent to both drugs and proteins.

To overcome these limitations, recent studies have explored multimodal integration to enhance predictive performance. MCL-DTI [27] extracts features from both drug molecule images and chemical text information that are then combined to form a multimodal drug representation fused with the target sequence for DTI prediction. In addition, MMDG-DTI [28] incorporates two complementary features: textual embeddings from pretrained language models, and structural embeddings derived from molecular graphs and protein sequence encoders. Despite the potential of multimodal integration, effectively optimizing these methods remains challenging, and they do not always outperform single-modality approaches in predictive accuracy.

Motivated by these challenges, we propose TriDTI, a novel framework that simultaneously leverages three distinct modalities for both drugs and proteins. Unlike prior approaches that rely on a single or dual representation, TriDTI incorporates structural, sequential, and relational features within a unified learning paradigm. Furthermore, cross-modal contrastive learning is employed to strengthen semantic alignment, and a dynamic fusion strategy adaptively balances modality contributions, enabling the capture of intricate DTI patterns often overlooked by previous models. Our contributions are summarized as follows:

- **Novel tri-modal framework:** TriDTI is a novel DTI prediction model to jointly utilize structural, sequential, and relational modalities for both drugs and proteins, expanding beyond the limitations of single- or dual-modality designs.
- **Enhanced modality alignment:** We design a projection layer combined with cross-modal contrastive learning to enforce semantic consistency both across instances and between modalities, addressing the challenges of joint optimization in multimodal learning.
- **Adaptive fusion:** We introduce a two-stage fusion mechanism in which soft attention dynamically weights modality-specific contributions and cross-attention models DTIs through interaction-aware representations, yielding more accurate DTI predictions.

## Materials and methods

**Figure 1:**
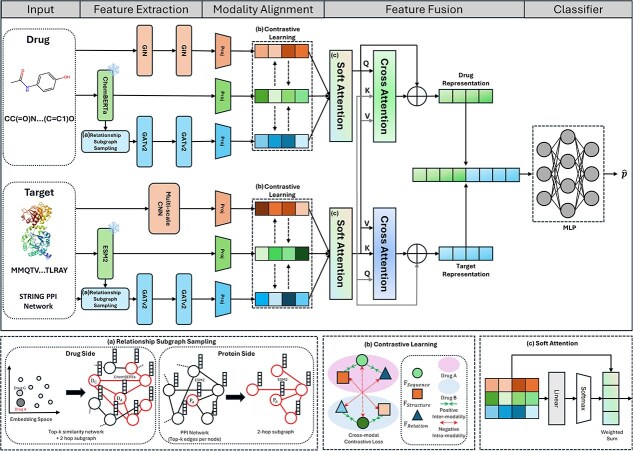
The overall architecture of TriDTI, which first extracts modality-specific features—including structural embeddings (graphs and CNNs), sequence-based representations (SMILES and amino acids), and network-derived relational features (subgraphs encoded with GATv2)—and projects them into a unified latent space for cross-modal contrastive alignment; this is followed by a two-stage fusion mechanism where soft attention adaptively balances each modality’s contribution while cross-attention models interaction-aware representations, eventually passing the fused output to a prediction layer to determine interaction probabilities.

TriDTI consists of four main stages: (i) feature extraction, (ii) modality alignment (iii) feature fusion, and (iv) classification. The overall architecture is shown in Fig. 1, and the details of each component are described in the following sections.

### Feature extraction

#### Structural feature

We explicitly encode the structural characteristics of drugs and proteins using graph and convolution architectures. Drug molecules are represented as graphs derived from their SMILES codes using RDKit, where atoms are nodes and bonds are edges. Each atom is encoded into a 79-dimensional feature vector encompassing properties such as atom type, bond degree, hydrogen count, implicit valence, and aromaticity. A 2-layer graph isomorphism network (GIN) is applied to capture the molecular topology:


1 \begin{align*}& h_{i}^{(k)} = MLP^{(k)}\left((1+\epsilon^{(k)})\cdot h_{i}^{(k-1)}+\sum_{j\in{\mathcal{N}(i)}}h_{j}^{(k-1)}\right)\end{align*}

where $h_{i}^{(k)}$ denotes the embedding of atom $i$ at layer $k$, $\mathcal{N}(i)$ is the neighbors of node $i$, and $\epsilon ^{(k)}$ is a learnable scalar. The final molecular representations are obtained by averaging the embeddings of all atoms in the last layer, forming the drug-level embedding matrix $Z_{{\textrm{ Structure}}_{D}}\in \mathbb{R}^{N_{d}\times d_{h_{1}}}$.

For proteins, we employ a multi-scale CNN to capture motifs of varying lengths from their amino acid sequences. The input sequences are first mapped to a learnable embeddings and passed through three parallel convolutional branches with kernel sizes of 1, 3, and 5, respectively. Each branch consists of three convolutional layers that refine local features. The outputs are then aggregated by AdaptiveMaxPooling to produce the protein embedding matrix $Z_{{\textrm{Structure}}_{T}}\in \mathbb{R}^{N_{t} \times d_{h_{2}}}$, encoding functional motifs and multi-scale dependencies.

#### Sequential feature

Sequence-based embeddings provide semantic and contextual features that complement explicit structures. Token-level embeddings from pretrained large language models (LLMs) are mean-pooled to obtain sequence-level representations. For drugs, we adopt ChemBERTa, trained on large SMILES corpora, which captures chemical grammar and higher-order molecular patterns. This produces a sequence embedding matrix $Z_{{\textrm{Sequence}}_{D}}\in \mathbb{R}^{{N_{d}}\times{h_{3}}}$.

For proteins, we use ESM2-t33-650M-UR50D [29], a transformer model with 650 M parameters trained on protein sequences. Its pooled embeddings form a matrix $Z_{{\textrm{Sequence}}_{T}}\in \mathbb{R}^{N_{t}\times{h_{4}}}$. These representations encode long-range dependencies relevant to folding and function. By anchoring on large-scale pretraining, these sequence-based representations offer stable and semantically rich priors for downstream modeling.

#### Relational feature

TriDTI captures relational information beyond individual entities by modeling dependencies within global interaction networks. This is achieved through relational subgraph sampling, a method that extracts relevant neighborhood topologies from drug–drug similarities and protein–protein interaction (PPI) networks to create localized representations.

The process for each entity is as follows. We first obtain node features for drugs from a pretrained LLM, ChemBERTa, denoted as $Z_{{\textrm{Sequence}}_{D}}$, and for proteins from ESM2, denoted as $Z_{{\textrm{Sequence}}_{T}}$. For drug entities, we construct a similarity network based on the cosine similarity of these $Z_{{\textrm{Sequence}}_{D}}$ embeddings. We then perform subgraph sampling by reducing the network density to retain only the top-$k$ edges and extracting 2-hop subgraphs. Similarly, for protein entities, we leverage the STRING PPI network [30] whose nodes are initialized with the $Z_{{\textrm{Sequence}}_{T}}$ embeddings. We sample subgraphs by applying the top-$k$ sparsification based on confidence scores and deriving 2-hop subgraphs.

Next, a 2-layer graph attention network version-2 (GATv2) [31] is applied to these subgraphs to aggregate relational information. The node update rule for the GATv2 is defined as:


2 \begin{align*}& h_{i}^{(k)} = \sigma\left(\sum_{j\in\mathcal{N}(i)} \alpha_{ij}^{(k)} W^{(k)}h_{j}^{(k-1)}\right),\end{align*}

where $h_{i}^{(k)}$ is the embedding of node $i$ at layer $k$, and attention weights $\alpha _{ij}^{(k)}$ are computed as:


3 \begin{align*}& \alpha_{ij}^{(k)} = \frac{e^{\left(a^\top\sigma (W^{(k)} [h_{i}^{(k-1)} \Vert h_{j}^{(k-1)}])\right)}}{\sum_{l\in\mathcal{N}(i)} e^{\left(a^\top\sigma (W^{(k)} [h_{i}^{(k-1)} \Vert h_{l}^{(k-1)}])\right)}}.\end{align*}

The final relation embeddings for drugs and proteins are obtained by averaging the node embeddings within their respective subgraphs, which follows the same formulation:


4 \begin{align*}& z_{{\textrm{Relation}}} = \tfrac{1}{|\mathcal{V}_{{\textrm{sub}}}|}\sum_{i\in\mathcal{V}_{{\textrm{sub}}}} h_{i}^{(2)}\end{align*}

where $\mathcal{V}_{{\textrm{sub}}}$ is the set of nodes in the sampled subgraph. Collecting these subgraph-level representations across all drugs and proteins yields the final relational embedding matrices $Z_{{\textrm{Relation}}_{D}} \in \mathbb{R}^{N_{d} \times d_{h_{3}}}$ and $Z_{{\textrm{Relation}}_{T}} \in \mathbb{R}^{N_{t} \times d_{h_{3}\!\!}}$. This formulation integrates local interaction patterns with global biological context, thereby complementing both structural and sequence-based features.

Unlike prior graph-based DTI frameworks [32, 33] that construct a unified heterogeneous biological network and perform end-to-end message passing across multiple entity types, TriDTI instead adopts a modular relational representation strategy. Relational information is encoded independently through localized subgraph representations derived from drug–drug similarity and PPI networks, rather than through joint propagation over a single heterogeneous graph. This design enables relational features to complement sequential and structural modalities without entangling heterogeneous propagation paths, facilitating more flexible multimodal fusion while reducing reliance on large, densely connected biological networks.

### Modality alignment

Effective integration of heterogeneous features from multiple modalities in TriDTI requires aligning embeddings in a shared latent space. Modality-specific projection networks are employed to map embeddings of varying dimensions into a unified space, ensuring both dimensional consistency and the ability to capture non-linear relationships. Formally, for a set of modality embeddings $Z_{M} \in \mathbb{R}^{N \times d_{m}}$, each embedding vector is transformed through a 2-layer feed-forward network with GELU activation:


5 \begin{align*}& \tilde{Z}_{M} = \textrm{GELU}(Z_{M}W_{1} + B_{1})W_{2} + B_{2},\end{align*}

To further ensure that embeddings from different modalities are semantically aligned, a bidirectional cross-modality contrastive learning objective is applied. In this framework, projected embeddings $\tilde{Z}_{M1}$ and $\tilde{Z}_{M2}$, form positive pairs for each entity $i$, while embeddings of different entities within the same modality serve as negatives. The directional loss from $M_{1}$ to $M_{2}$ is defined as:


6 \begin{align*}& \mathcal{L}_{M_{1} \rightarrow M_{2}} = \sum_{n=1}^{N} \sum_{i=1}^{B} - \log \frac{e^{(\textrm{sim}(\tilde{Z}_{{M_{1}},i}^{(n)}, \tilde{Z}_{{M_{2}},i}^{(n)})/\tau)}} {\sum_{j=1}^{B} e^{(\textrm{sim}(\tilde{Z}_{{M_{1}},i}^{(n)}, \tilde{Z}_{{M_{1}},j}^{(n)})/\tau)}},\end{align*}

where $N$ is the number of mini-batches, $B$ is the mini-batch size, $\textrm{sim}(\cdot )$ denotes cosine similarity, and $\tau $ is a temperature hyperparameter. The bidirectional loss


7 \begin{align*}& \mathcal{L}_{M_{1},M_{2}} = \frac{1}{2}\left(\mathcal{L}_{M_{1} \rightarrow M_{2}} + \mathcal{L}_{M_{2} \rightarrow M_{1}}\right)\end{align*}

ensures symmetric alignment between modalities. The final contrastive loss is computed over selected modality pairs for both drugs and targets:


8 \begin{align*}& \begin{split} \mathcal{L}_{\textrm{contrast}} = \;& \mathcal{L}_{(Sequence:{D}, Structure_{D})} + \mathcal{L}_{(Sequence:{D}, Relation_{D})} \\ &+ \mathcal{L}_{(Sequence:{T}, Structure_{T})} + \mathcal{L}_{(Sequence:{T}, Relation_{T})}, \end{split}\end{align*}

focusing on aligning other modalities to the pretrained sequential representations. By encouraging closeness among embeddings of the same entity across modalities while separating embeddings of different entities within each modality, this modality alignment step promotes consistent, discriminative, and semantically coherent representations across the tri-modal feature space, enhancing the predictive capability of TriDTI.

### Feature fusion

TriDTI employs a two-stage attention-based fusion strategy to integrate heterogeneous modality embeddings of drugs and proteins. This approach balances modality-specific strengths while mitigating redundancy and noise, yielding interaction-specific representations that capture both entity-level and pair-level dependencies.

First, a soft attention module adaptively weighs the contribution of each modality. Given modality features $\tilde{Z}_{m}$ for an entity, the attention scores $\alpha _{m}$ are computed using a two-layer multi-layer perceptron (MLP) with Tanh activation, and normalized across modalities via a softmax function. The fused entity representation is then obtained as a weighted sum of modality embeddings:


9 \begin{align*}& Z_{\textrm{Soft}} = \sum_{m} \alpha_{m} \tilde{Z}_{m}.\end{align*}

Second, the fused drug and protein embeddings are refined through a bidirectional cross-attention module. In this design, the query $Q$ originates from one entity, while the key $K$ and value $V$ are projected from the other, enabling each entity to selectively attend to features of its counterpart. Formally, the cross-attention from drug to protein is defined as


10 \begin{align*}& \textrm{Attn}(D \rightarrow T) = \textrm{FFNN}\left(\textrm{softmax}\left(\frac{Q_{D} K_{T}^\top}{\sqrt{d}}\right)V_{T}\right),\end{align*}

with a symmetric formulation for $\textrm{Attn}(T \rightarrow D)$. Residual connections are then applied to preserve entity-specific information while incorporating complementary interaction cues, leading to the final embeddings:


11 \begin{align*} & Z_{{\textrm{Final}}_{D}} = Z_{{\textrm{Soft}}_{D}} + \textrm{Attn}(D \rightarrow T), \end{align*}

12 \begin{align*} & Z_{{\textrm{Final}}_{T}} = Z_{{\textrm{Soft}}_{T}} + \textrm{Attn}(T \rightarrow D). \end{align*}

Here, $Z_{{\textrm{Final}}_{D}}$ and $Z_{{\textrm{Final}}_{T}}$ serve as the final drug and protein representations, simultaneously retaining modality integrated features and cross-entity contextual information, which form the basis for downstream interaction prediction.

### Classification

The final representations of drugs and proteins, enhanced by the bidirectional cross-attention module, are combined to predict the probability of interaction. Specifically, the two vectors $Z^{{\textrm{Final}}_{D}}$ and $Z^{{\textrm{Final}}_{T}}$ are concatenated to form a unified representation $Z^{{\textrm{Final}}_{\textrm{DTI}}}$, which is then fed into an MLP-based classifier. The classifier consists of multiple fully connected layers interleaved with GELU activation functions and dropout regularization, enabling it to capture complex nonlinear dependencies between drugs and proteins. Formally, the prediction is obtained as


13 \begin{align*}& \hat{y} = \sigma\left(MLP(Z_{{\textrm{Final}}_{\textrm{DTI}}})\right)\end{align*}

where $\hat{y}\in{[0, 1]}$ denotes the predicted interaction probability, and $\sigma (\cdot )$ is the sigmoid activation function.

### Overall loss function

To optimize both prediction accuracy and modality consistency, the model is trained with a composite loss function that combines binary cross-entropy (BCE) loss and cross-modality contrastive loss. The BCE loss directly supervises DTI prediction by minimizing the discrepancy between the predicted probability $\hat{y}$ and the ground-truth label $y$:


14 \begin{align*}& \mathcal{L}_{\textrm{BCE}} = -y\log(\hat{y}) - (1-y)\log(1-\hat{y}).\end{align*}

The total loss is defined as a weighted sum of BCE loss and the previously defined contrastive loss:


15 \begin{align*}& \mathcal{L}_{\textrm{total}} = \mathcal{L}_{\textrm{BCE}} + \lambda \mathcal{L}_{\textrm{contrast}},\end{align*}

where $\lambda $ is a hyperparameter that balances prediction accuracy and modality alignment. In our experiments, we set $\lambda = 1 \times 10^{-4}$ to provide a small but effective regularization from the contrastive objective. This joint optimization encourages the model not only to maximize predictive performance but also to maintain semantic consistency across heterogeneous modalities, thereby enhancing both generalization and representation quality.

## Results

### Datasets

We employed three publicly available benchmark datasets for evaluation: DAVIS [34], BioSNAP [35], and DrugBank [36]. The DAVIS dataset consists of 68 drugs and 379 target proteins, providing experimental measurements of drug–target binding affinities. Following prior work, we binarized the affinity values by treating drug–target pairs with dissociation constant ($K_{d}$) values below 30 as positive interactions and all others as negative, thus reformulating the task into a binary classification problem. For BioSNAP and DrugBank, we used the preprocessed versions from MolTrans [37] and HyperAttentionDTI [15], respectively. In these versions, drug–target pairs were extracted from the original datasets, and negative sampling was applied to ensure a $\sim $1:1 ratio of positive to negative interactions. To maintain data integrity, we further removed drug samples with invalid SMILES strings that could not be converted into molecular graphs.

To incorporate relational knowledge, we leveraged the PPI dataset from STRING [30] that provides probabilistic confidence scores for functional associations between proteins. Using STRING PPIs, we constructed separate PPI networks for each benchmark by including only the proteins present in the corresponding DTI dataset. This approach ensures that the relational information is specific to each benchmark while capturing the functional associations relevant to the modeled proteins. These networks were subsequently integrated as an additional modality input to our model. The statistics of the resulting experimental datasets are summarized in Table 1.

**Table 1:** Statistics of the benchmark datasets for our experiments.

**Dataset**	**Drugs**	**Targets**	**DTIs**		**PPIs**
			**Positive**	**Negative**	
DAVIS	68	379	1506	9597	15 734
BioSNAP	4502	2181	13 811	13 622	193 212
DrugBank	6645	4254	17 511	17 511	237 405

### Experimental settings

For a robust assessment, we adopted five-fold cross-validation. Each dataset was split into training, validation and test sets in a 7:1:2 ratio. Model performance was evaluated using four standard metrics: area under the receiver operating characteristic curve (AUROC), area under the precision–recall curve (AUPRC), F1 score, and accuracy. Training was conducted using the AdamW optimizer with a learning rate of 5e-4, a batch size of 16, and a dropout rate of 0.1 for up to 100 epochs. Analysis of the training dynamics (see Supplementary Section S4) confirmed that the model consistently converged within this epoch limit, demonstrating stable optimization. The model parameters achieving the highest AUROC on the validation set were selected for reporting final test results. Detailed hyperparameter configurations for TriDTI are provided in Table 2, and a sensitivity analysis of the modality alignment hyperparameters ($\tau $ and $\lambda $) is presented in Supplementary Table S1. To ensure a fair and reproducible comparison, all baseline models were rigorously trained, validated, and tested using the identical data splits employed for TriDTI. For model implementations, we adhered to the hyperparameters and configurations explicitly reported in the original work. Where details were unavailable or incompatible with our datasets, hyperparameters were empirically tuned to reflect the scale and characteristics of each dataset.

**Table 2:** Hyperparameter configurations for TriDTI across DAVIS, BIOSNAP, and DrugBank datasets.

**Hyperparameter**	**DAVIS**	**BIOSNAP**	**DrugBank**
*Structural feature*			
GIN input dim	79	79	79
GIN output dim ($h_{1}$)	128	64	64
CNN input dim	128	64	64
CNN output dim ($h_{2}$)	128	64	64
*Sequential feature*			
ChemBERTa input dim	510	510	510
ChemBERTa output dim ($h_{3}$)	768	768	768
ESM2 input dim	1024	1024	1024
ESM2 output dim ($h_{4}$)	1280	1280	1280
*Relational feature*			
Drug GATv2 hidden dim ($h_{5}$)	128	64	64
Target GATv2 hidden dim ($h_{5}$)	128	64	64
*Modality alignment*			
Projection dim	(128, 128)	(128, 64)	(256, 64)
Contrastive temperature ($\tau $)	0.1	0.1	0.1
Contrastive weight ($\lambda $)	0.0001	0.0001	0.0001
*Modality fusion*			
Soft attention hidden dim	(128, 3)	(64, 3)	(64, 3)
Cross-attention output dim	128	64	64
Cross-attention num heads	8	8	8

### Performance evaluation

TriDTI consistently achieved the best performance among all existing state-of-the-art models across all three benchmark datasets, as summarized in Table 3. On the DAVIS dataset, TriDTI recorded an AUROC of 0.9391 and an AUPRC of 0.7605, corresponding to relative improvements of 0.24% and 0.88% over the previous best-performing model, GPS-DTI. While MGMA-DTI reported a higher F1 score, its performance across the other metrics did not generalize as well. In contrast, TriDTI demonstrated a uniformly strong and balanced predictive capability across all other major evaluation metrics, recording a high accuracy of 0.9234.

**Table 3:** DTI prediction performance on DAVIS, BioSNAP, and DrugBank datasets, where values indicate the mean and standard deviation over five-fold cross-validation.

**Dataset**	**Methods**	**AUROC**	**AUPRC**	**F1**	**Accuracy**
**DAVIS**	TransformerCPI	0.8399 $\pm $ 0.0125	0.5329 $\pm $ 0.0066	0.5141 $\pm $ 0.0394	0.8723 $\pm $ 0.0073
	MGraphDTA	0.9211 $\pm $ 0.0118	0.7064 $\pm $ 0.0163	0.6843 $\pm $ 0.0160	0.9087 $\pm $ 0.0053
	HyperAttentionDTI	0.9221 $\pm $ 0.0108	0.7214 $\pm $ 0.0133	0.6911 $\pm $ 0.0168	0.9184 $\pm $ 0.0024
	MCL-DTI	0.8967 $\pm $ 0.0114	0.7050 $\pm $ 0.0241	0.6660 $\pm $ 0.0225	0.9180 $\pm $ 0.0057
	DLM-DTI	0.9290 $\pm $ 0.0114	0.7436 $\pm $ 0.0249	0.7083 $\pm $ 0.0203	0.9194 $\pm $ 0.0058
	MMDG-DTI	0.9166 $\pm $ 0.0058	0.7155 $\pm $ 0.0242	0.6848 $\pm $ 0.0134	0.9094 $\pm $ 0.0068
	MGMA-DTI	0.8937 $\pm $ 0.0072	0.6735 $\pm $ 0.0252	**0.8311 $\pm $ 0.0102**	0.8212 $\pm $ 0.0354
	GPS-DTI	0.9368 $\pm $ 0.0069	0.7538 $\pm $ 0.0138	0.7245 $\pm $ 0.0129	**0.9244 $\pm $ 0.0048**
	TriDTI	**0.9391 $\pm $ 0.0031**	**0.7605 $\pm $ 0.0114**	0.7186 $\pm $ 0.0100	0.9234 $\pm $ 0.0014
**BioSNAP**	TransformerCPI	0.8714 $\pm $ 0.0040	0.8773 $\pm $ 0.0050	0.7977 $\pm $ 0.0038	0.7877 $\pm $ 0.0097
	MGraphDTA	0.9049 $\pm $ 0.0026	0.9117 $\pm $ 0.0030	0.8316 $\pm $ 0.0029	0.8263 $\pm $ 0.0035
	HyperAttentionDTI	0.9122 $\pm $ 0.0035	0.9181 $\pm $ 0.0041	0.8410 $\pm $ 0.0053	0.8391 $\pm $ 0.0072
	MCL-DTI	0.8773 $\pm $ 0.0025	0.8788 $\pm $ 0.0037	0.8079 $\pm $ 0.0049	0.8060 $\pm $ 0.0043
	DLM-DTI	0.9115 $\pm $ 0.0031	0.9158 $\pm $ 0.0025	0.8420 $\pm $ 0.0068	0.8418 $\pm $ 0.0051
	MMDG-DTI	0.9093 $\pm $ 0.0022	0.9149 $\pm $ 0.0035	0.8393 $\pm $ 0.0021	0.8345 $\pm $ 0.0023
	MGMA-DTI	0.8905 $\pm $ 0.0040	0.8946 $\pm $ 0.0069	0.8180 $\pm $ 0.0052	0.8131 $\pm $ 0.0083
	GPS-DTI	0.9256 $\pm $ 0.0039	0.9259 $\pm $ 0.0056	0.8594 $\pm $ 0.0057	0.8555 $\pm $ 0.0068
	TriDTI	**0.9274 $\pm $ 0.0030**	**0.9280 $\pm $ 0.0029**	**0.8605 $\pm $ 0.0039**	**0.8567 $\pm $ 0.0067**
**DrugBank**	TransformerCPI	0.8451 $\pm $ 0.0051	0.8480 $\pm $ 0.0071	0.7729 $\pm $ 0.0035	0.7679 $\pm $ 0.0031
	MGraphDTA	0.8780 $\pm $ 0.0042	0.8823 $\pm $ 0.0063	0.8032 $\pm $ 0.0039	0.7948 $\pm $ 0.0073
	HyperAttentionDTI	0.8878 $\pm $ 0.0035	0.8922 $\pm $ 0.0046	0.8112 $\pm $ 0.0036	0.8066 $\pm $ 0.0052
	MCL-DTI	0.8450 $\pm $ 0.0032	0.8435 $\pm $ 0.0051	0.7762 $\pm $ 0.0038	0.7733 $\pm $ 0.0037
	DLM-DTI	0.8990 $\pm $ 0.0051	0.9008 $\pm $ 0.0034	0.8238 $\pm $ 0.0074	0.8181 $\pm $ 0.0132
	MMDG-DTI	0.8768 $\pm $ 0.0179	0.8760 $\pm $ 0.0225	0.8064 $\pm $ 0.0133	0.7934 $\pm $ 0.0171
	MGMA-DTI	0.8676 $\pm $ 0.0036	0.8693 $\pm $ 0.0107	0.7944 $\pm $ 0.0033	0.7826 $\pm $ 0.0075
	GPS-DTI	0.9120 $\pm $ 0.0019	0.9101 $\pm $ 0.0029	0.8431 $\pm $ 0.0039	0.8395 $\pm $ 0.0049
	TriDTI	**0.9182 $\pm $ 0.0042**	**0.9180 $\pm $ 0.0068**	**0.8477 $\pm $ 0.0036**	**0.8458 $\pm $ 0.0037**

Note: The best and second-best results are shown in bold and underline, respectively.

The advantage of TriDTI is further substantiated on the BioSNAP and DrugBank datasets, where its overall superiority is more pronounced. For BioSNAP, TriDTI achieved the highest results across all four metrics: AUROC (0.9274), AUPRC (0.9280), F1 score (0.8605), and accuracy (0.8567). Similarly, TriDTI obtained the best performance on DrugBank recording AUROC 0.9182, AUPRC 0.9180, F1 score 0.8477, and accuracy 0.8458. When compared against the average performance of all other baseline models, these results demonstrate a more substantial margin of improvement. For instance, TriDTI surpasses the average AUROC and AUPRC of all competing models by 2.92% and 2.52% on BioSNAP, and 4.55%, 4.38% on DrugBank, respectively. These results highlight the effectiveness of TriDTI’s modality-integrated representation learning, achieving superior and consistent performance across diverse datasets.

### Ablation study

**Figure 2:**
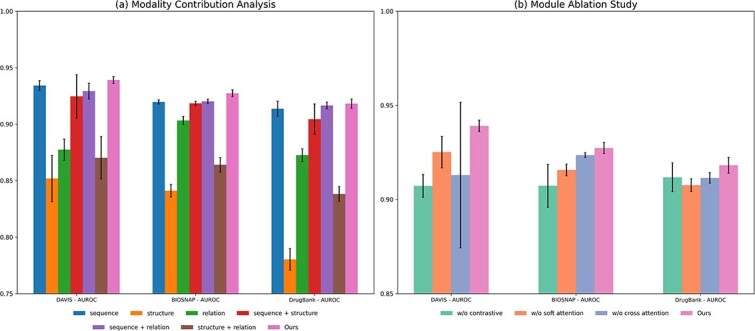
Ablation study results of TriDTI on the DAVIS, BioSNAP, and DrugBank datasets. The figure presents two comparative analyses: (a) Modality contribution analysis assesses the contribution of individual feature sources by comparing the full model against variants where a single or dual input modality is excluded. (b) Module ablation study validates the functional necessity of core architectural units by comparing the full model against variants excluding each modular component. Bars represent the mean and standard deviation over five-fold cross-validation, reported by AUROC.

We further analyzed the contribution of individual modalities and the importance of key components in the TriDTI. By systematically removing specific modalities or architectural modules, we evaluated how each element influenced the overall predictive performance. The experimental results are summarized in Fig. 2.

#### Modality contribution analysis

The contribution of each modality was analyzed by comparing single-, dual-, and tri-modality configurations. Among single-modality settings, the sequence-only model consistently achieved the best performance across all datasets, whereas relational and structural modalities exhibited relatively lower accuracy. This finding highlights sequence-based semantic information from pretrained language models as the most informative signal for DTI prediction.

Models that included the sequence modality generally maintained strong performance, indicating its robustness across different datasets. However, performance gains were not always guaranteed when two modalities were combined. In several cases, dual-modality models underperformed the sequence-only baseline, suggesting that naive feature fusion does not necessarily lead to improved predictions. Notable exceptions were observed for BioSNAP and DrugBank, where integrating sequence and relational modalities yielded performance improvements, implying complementary contributions from relational information. In contrast, the joint utilization of all three modalities consistently improved performance across all datasets. This outcome demonstrates that full multimodal integration enables TriDTI to capture complementary information beyond what is accessible through single or limited dual-modality configurations. In addition, the soft attention weights offered insight into how the model adaptively emphasizes different modalities based on dataset characteristics (see Supplementary Section S2).

#### Module ablation study

To validate the necessity of the proposed architecture, we assessed the functional role of TriDTI’s core modules by comparing the full framework against various ablated variants. Across all datasets, the complete model consistently outperformed its ablated variants, confirming the effectiveness of the proposed design. Removing the contrastive learning module resulted in a performance degradation of 2.09% on average. This degradation shows that explicit cross-modal alignment is crucial for learning robust multimodal embeddings, as its absence hinders the model’s ability to fully exploit the complementary nature of heterogeneous features. Furthermore, as shown in Supplementary Section S4, analysis of the training dynamics confirmed that the contrastive objective led to enhanced convergence stability and superior validation AUROC.

The attention-based fusion mechanism was also validated through its components. Excluding the soft attention module reduced performance by 1.30% on average, suggesting that selectively emphasizing informative features within each modality contributes to improving prediction accuracy. A comparable performance drop of 1.31% on average resulted from the removal of the cross-attention module. This result emphasizes the benefit of modeling pairwise interactions at the drug–target level. Overall, the ablation results confirmed that each architectural component meaningfully contributes to the final performance, and that combining contrastive alignment with attention-based fusion is crucial for effective multimodal integration in TriDTI.

### Model interpretability

**Figure 3:**
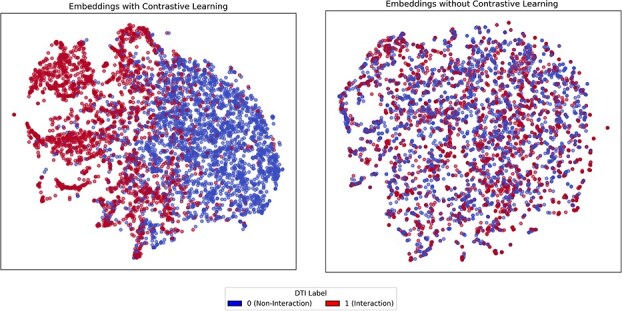
t-SNE visualization of joint drug–target embeddings. The left panel shows embeddings obtained from the model trained with contrastive learning, while the right panel corresponds to embeddings learned without the contrastive objective. Embeddings learned with contrastive learning exhibit more clearly separated and structured clusters between interaction and non-interaction samples, indicating enhanced discriminative representation learning compared with the non-contrastive counterpart.

Contrastive learning plays an important role in shaping the quality of the representation space. Figure 3 presents t-SNE visualizations of the joint drug–target embeddings produced by TriDTI on the BioSNAP dataset, comparing models trained with and without the contrastive learning objective. As illustrated in the figure, embeddings generated with contrastive learning for more clearly separated and structured clusters corresponding to interaction and noninteraction labels. In contrast, embeddings obtained without contrastive learning show substantial overlap between classes, indicating reduced discriminative capability. These observations suggest that contrastive learning guides the model to organize the representation space in a way that better captures underlying DTI patterns. Furthermore, detailed analysis and visualization of the bi-directional Cross-Attention mechanism (see Supplementary Section S3) confirmed that the model learns robust and mutual interaction representations by exhibiting complementary attention patterns.

### Cold-start settings

**Table 4:** DTI prediction performance comparison on DAVIS under three cold-start scenarios: Unseen Drug, Unseen Target, and Unseen Binding, where values represent the mean and standard deviation over five-fold cross-validation.

Model	Unseen Drug		Unseen Target		Unseen Binding	
	AUROC	AUPRC	AUROC	AUPRC	AUROC	AUPRC
TransformerCPI	**0.7483 $\pm $ 0.0304**	0.3470 $\pm $ 0.0789	0.7972 $\pm $ 0.0342	0.4546 $\pm $ 0.0961	0.7212 $\pm $ 0.0575	0.3086 $\pm $ 0.1324
MGraphDTA	0.7230 $\pm $ 0.0450	0.3554 $\pm $ 0.0966	0.8492 $\pm $ 0.0432	0.5314 $\pm $ 0.1315	0.6314 $\pm $ 0.0775	0.2028 $\pm $ 0.0691
HyperAttentionDTI	0.7400 $\pm $ 0.0297	0.3676 $\pm $ 0.1100	0.8714 $\pm $ 0.0292	0.5955 $\pm $ 0.0986	0.6525 $\pm $ 0.0777	0.2656 $\pm $ 0.1165
MCL-DTI	0.7260 $\pm $ 0.0376	0.3446 $\pm $ 0.0865	0.7871 $\pm $ 0.0391	0.4477 $\pm $ 0.0979	0.6674 $\pm $ 0.0776	0.2530 $\pm $ 0.1316
DLM-DTI	0.7313 $\pm $ 0.0414	**0.3861 $\pm $ 0.0918**	0.8247 $\pm $ 0.0605	0.5334 $\pm $ 0.1519	0.7016 $\pm $ 0.0808	0.2902 $\pm $ 0.0452
MMDG-DTI	0.7409 $\pm $ 0.0852	0.3748 $\pm $ 0.1159	0.8529 $\pm $ 0.0383	0.5474 $\pm $ 0.1067	0.6490 $\pm $ 0.1232	0.2665 $\pm $ 0.1509
MGMA-DTI	0.7420 $\pm $ 0.0489	0.3746 $\pm $ 0.0738	0.7260 $\pm $ 0.0545	0.3883 $\pm $ 0.0876	0.5729 $\pm $ 0.1287	0.1977 $\pm $ 0.0944
GPS-DTI	0.6904 $\pm $ 0.0503	0.3318 $\pm $ 0.0430	0.8870 $\pm $ 0.0255	0.6280 $\pm $ 0.1019	0.6931 $\pm $ 0.0432	0.2597 $\pm $ 0.0239
TriDTI	0.7302 $\pm $ 0.0357	0.3345 $\pm $ 0.0717	**0.8923 $\pm $ 0.0297**	**0.6328 $\pm $ 0.0896**	**0.7909 $\pm $ 0.0488**	**0.4202 $\pm $ 0.0712**

Note: The best and second-best results are shown in bold and underline, respectively.

**Table 5:** DTI prediction performance comparison on BioSNAP under three cold-start scenarios: Unseen Drug, Unseen Target, and Unseen Binding, where values represent the mean and standard deviation over five-fold cross-validation.

Model	Unseen Drug		Unseen Target		Unseen Binding	
	AUROC	AUPRC	AUROC	AUPRC	AUROC	AUPRC
TransformerCPI	0.8661 $\pm $ 0.0094	0.8768 $\pm $ 0.0071	0.7267 $\pm $ 0.0366	0.7477 $\pm $ 0.0510	0.7040 $\pm $ 0.0618	0.7262 $\pm $ 0.0809
MGraphDTA	0.8571 $\pm $ 0.0089	0.8735 $\pm $ 0.0067	0.7652 $\pm $ 0.0268	0.7907 $\pm $ 0.0381	0.6866 $\pm $ 0.0608	0.7244 $\pm $ 0.0702
HyperAttentionDTI	0.8694 $\pm $ 0.0104	0.8838 $\pm $ 0.0092	0.7868 $\pm $ 0.0219	0.8214 $\pm $ 0.0254	0.7065 $\pm $ 0.0596	0.7473 $\pm $ 0.0721
MCL-DTI	0.8150 $\pm $ 0.0164	0.8321 $\pm $ 0.0097	0.7168 $\pm $ 0.0271	0.7447 $\pm $ 0.0437	0.6399 $\pm $ 0.0417	0.6749 $\pm $ 0.0700
DLM-DTI	0.8266 $\pm $ 0.0538	0.8492 $\pm $ 0.0431	0.8388 $\pm $ 0.0138	0.8552 $\pm $ 0.0209	0.7213 $\pm $ 0.0655	0.7550 $\pm $ 0.0818
MMDG-DTI	0.8691 $\pm $ 0.0105	0.8856 $\pm $ 0.0081	0.8104 $\pm $ 0.0171	0.8339 $\pm $ 0.0125	0.7503 $\pm $ 0.0554	0.7852 $\pm $ 0.0661
MGMA-DTI	0.8660 $\pm $ 0.0079	0.8745 $\pm $ 0.0083	0.6689 $\pm $ 0.0292	0.6904 $\pm $ 0.0435	0.6388 $\pm $ 0.0445	0.6651 $\pm $ 0.0684
GPS-DTI	0.8735 $\pm $ 0.0156	0.8825 $\pm $ 0.0166	**0.8684 $\pm $ 0.0122**	**0.8804 $\pm $ 0.0198**	0.7882 $\pm $ 0.0446	**0.8110 $\pm $ 0.0581**
TriDTI	**0.8834 $\pm $ 0.0108**	**0.8899 $\pm $ 0.0135**	0.8670 $\pm $ 0.0073	0.8750 $\pm $ 0.0220	**0.7983 $\pm $ 0.0305**	0.8080 $\pm $ 0.0395

Note: The best and second-best results are shown in bold and underline, respectively.

A cold-start scenario, where a model encounters previously unseen drugs, targets, or binding pairs, constitutes one of the most challenging settings in DTI prediction. Under these settings, TriDTI demonstrated strong performance across the DAVIS, BioSNAP, and DrugBank datasets, as summarized in Tables 4–6. In the Unseen Drug setting, TriDTI showed comparatively lower performance on the DAVIS dataset than some baseline methods. However, it achieved the best results on both BioSNAP and DrugBank in terms of AUROC and AUPRC, suggesting effective generalization to previously unseen compounds in larger and more diverse chemical spaces. In the Unseen Target and Unseen Binding settings, TriDTI consistently ranked among the top two methods across all datasets, demonstrating robust generalization under diverse cold-start conditions. In particular, GPS-DTI exhibited notably strong performance in the Unseen Target scenario, which is likely attributable to its reliance on large-scale pretrained protein representations from ESM2. Overall, these results indicate that TriDTI is well suited for real-world DTI prediction scenarios, where new compounds and targets are continuously introduced.

**Table 6:** DTI prediction performance comparison on DrugBank under three cold-start scenarios: Unseen Drug, Unseen Target, and Unseen Binding, where values represent the mean and standard deviation over five-fold cross-validation.

Model	Unseen Drug		Unseen Target		Unseen Binding	
	AUROC	AUPRC	AUROC	AUPRC	AUROC	AUPRC
TransformerCPI	0.7674 $\pm $ 0.0322	0.3572 $\pm $ 0.0761	0.7240 $\pm $ 0.0159	0.7295 $\pm $ 0.0158	0.6892 $\pm $ 0.0098	0.6860 $\pm $ 0.0251
MGraphDTA	0.8316 $\pm $ 0.0095	0.8407 $\pm $ 0.0108	0.7573 $\pm $ 0.0053	0.7839 $\pm $ 0.0025	0.6911 $\pm $ 0.0062	0.7030 $\pm $ 0.0165
HyperAttentionDTI	0.8335 $\pm $ 0.0052	0.8426 $\pm $ 0.0049	0.7814 $\pm $ 0.0202	0.8091 $\pm $ 0.0164	0.6970 $\pm $ 0.0331	0.6950 $\pm $ 0.0463
MCL-DTI	0.7596 $\pm $ 0.0172	0.7729 $\pm $ 0.0107	0.6619 $\pm $ 0.0164	0.6796 $\pm $ 0.0134	0.5646 $\pm $ 0.0173	0.5585 $\pm $ 0.0314
DLM-DTI	0.8478 $\pm $ 0.0117	0.8514 $\pm $ 0.0117	0.8372 $\pm $ 0.0107	0.8461 $\pm $ 0.0107	0.7579 $\pm $ 0.0056	0.7615 $\pm $ 0.0104
MMDG-DTI	0.8332 $\pm $ 0.0194	0.8397 $\pm $ 0.0196	0.7780 $\pm $ 0.0374	0.7953 $\pm $ 0.0334	0.7071 $\pm $ 0.0154	0.7219 $\pm $ 0.0281
MGMA-DTI	0.8284 $\pm $ 0.0103	0.8346 $\pm $ 0.0124	0.6919 $\pm $ 0.0244	0.7011 $\pm $ 0.0229	0.6318 $\pm $ 0.0174	0.6183 $\pm $ 0.0197
GPS-DTI	0.8487 $\pm $ 0.0074	0.8572 $\pm $ 0.0040	**0.8681 $\pm $ 0.0155**	**0.8776 $\pm $ 0.0155**	0.7774 $\pm $ 0.0226	0.7841 $\pm $ 0.0248
TriDTI	**0.8688 $\pm $ 0.0086**	**0.8717 $\pm $ 0.0058**	0.8664 $\pm $ 0.0106	0.8725 $\pm $ 0.0102	**0.7943 $\pm $ 0.0161**	**0.7913 $\pm $ 0.0239**

Note: The best and second-best results are shown in bold and underline, respectively.

### Case study

The cold start analysis demonstrated TriDTI’s strong generalization ability to unseen data. However, this case study aims to highlight the practical utility of the model for real-world drug discovery. To validate our predictions for unknown DTIs, we used the DrugBank dataset. We first filtered all known drug–target pairs and then used the remaining candidate pool as input for our model. This process yielded a list of the 10 most promising novel candidates. After excluding a pair that lacked a 3D PDB structure, we subjected the remaining nine candidates to molecular docking simulations for validation.

**Figure 4:**
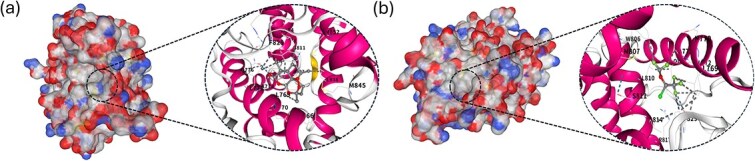
Molecular docking analysis of top-ranked pairs predicted by TriDTI. (a) Highest-ranked binding prediction: DB11638 interacting with P08235. (b) Second-ranked binding prediction: DB00753 interacting with P08235.

To further substantiate our predictions, we used the CB-Dock2 [38] docking server to compute Vina scores for the nine candidates. The detailed docking results, including the Vina score, cavity volume, center coordinates, and docking size for each pair, are presented in Table 7. The results showed that every pair yielded a binding affinity score of < −5 kcal/mol. In docking analysis, a Vina score below −5 kcal/mol is generally considered a strong indicator of potential DTI, with more negative values suggesting a more robust binding ability. The docking outcomes for the top two candidates are further visualized in Fig. 4 that shows their binding poses and key interactions with the target proteins.

**Table 7:** Top 9 docking results of drug–protein pairs selected by TriTDI

Drug ID	Protein ID	Vina score	Cavity volume	Center (x, y, z)	Docking size (x, y, z)
DB11638	P08235	−7.6	458	64, 58, −2	18, 18, 18
DB00753	P08235	−5.3	436	122, 24, 22	16, 16, 16
DB00637	P08913	−10.7	6	−5, −12, 10	26, 26, 26
DB07973	P08913	−9.6	6	−5, −12, 10	23, 23, 23
DB06144	P08913	−9.9	6	−5, −12, 10	25, 25, 25
DB01043	P08235	−7.0	436	122, 24, 22	16, 16, 16
DB05422	P08913	−9.4	6	−5, −12, 10	24, 24, 24
DB08685	P34903	−5.8	3772	142, 102, 133	28, 29, 35
DB05316	P08913	−9.7	6	−5, −12, −10	25, 25, 25

It should be noted that docking scores alone do not constitute experimental validation of DTIs. Rather, these results provide supportive, structure-based evidence that the model-predicted pairs are physically plausible and merit further investigation. Taken together, this case study demonstrates that TriDTI can effectively prioritize candidate drug–target pairs that are favorable for downstream structure-based analysis, thereby serving as a useful computational screening tool in the early stages of drug discovery.

## Conclusion

In this study, we present TriDTI, a novel deep learning framework designed to address the limitations of traditional DTI prediction models. The model simultaneously integrates three complementary modalities for both drugs and proteins: sequential representations from LLMs, structural features from molecular graphs and amino acid sequences, and relational information from biological networks. To balance the contributions of these heterogeneous modalities, we adopt a cross-modal contrastive learning strategy that enhances semantic alignment across feature spaces. In addition, a dynamic attention-based fusion mechanism is introduced to maximize predictive accuracy by adaptively weighting modality-specific contributions and modeling DTI patterns. Extensive experiments demonstrate that TriDTI consistently achieves the best performance across three benchmark datasets. Moreover, validation under cold-start scenarios and molecular docking case studies highlights its strong generalization capacity and practical utility in discovering novel drug–target pairs.

Although TriDTI is a useful tool for DTI prediction, several avenues remain for future exploration. First, while our current design incorporates pretrained LLM-based features, pretraining the molecular graph modality on large-scale datasets [39, 40] could further alleviate the imbalance among heterogeneous modalities and enhance structural representations. Second, although TriDTI effectively utilizes relational features through drug–drug similarities and PPIs, it currently does not rely on a comprehensive heterogeneous biological information network containing multiple entity types (e.g. diseases or side-effects). A promising direction involves augmenting the relational modality by incorporating such comprehensive networks and leveraging advanced heterogeneous graph representation learning methods [41, 42]. Third, TriDTI does not yet incorporate explicit 3D structural data, despite employing CNNs to model 1D protein sequences [43, 44]. Therefore, integrating 3D conformational information, potentially through geometric deep learning or structure-informed representations, would allow us to capture spatial interaction patterns more effectively. Fourth, the framework can be extended to integrate additional complementary modalities for both drugs and proteins, such as molecular images or textual descriptions, to achieve an even richer multimodal representation. Such extensions will further strengthen TriDTI’s capability and establish it as an even more versatile tool for advancing computational drug discovery.

Key Points

- We propose TriDTI, a novel tri-modal framework that integrates structural, sequential, and relational modalities to learn comprehensive representations by capturing diverse features of both drugs and proteins.
- The model employs a cross-modal contrastive learning strategy to enforce semantic alignment across disparate embedding spaces, effectively minimizing information loss during the integration of heterogeneous features.
- A two-stage adaptive fusion mechanism, combining soft attention and cross-attention, is designed to dynamically balance modality contributions and precisely model interaction-aware representations.

## Supplementary Material

bbag034_Supplemental_File

## Data Availability

The codes and datasets are available online at https://github.com/knhc1234/TriDTI.
