# Supplementary material for: TriDTI: tri-modal representation learning with cross-modal alignment for drug–target interaction prediction
Source: Brief Bioinform. 2026 Feb 5;27(1):bbag034. doi: 10.1093/bib/bbag034 (PMC12874921; doi:10.1093/bib/bbag034)
Supplement: bbag034_Supplemental_File [file bbag034_supplemental_file.docx]

**Supplementary Materials for:**

**TriDTI: Tri-modal Representation Learning with Cross-Modal Alignment for Drug-Target Interaction Prediction**

Gwang-Hyeon Yun^1^, Jong-Hoon Park^1^ and Young-Rae Cho^1,2,*^

^1^Division of Software, Yonsei University Mirae Campus, 1 Yeonsedae-gil, 26493, Gangwon-do, Republic of Korea

^2^Division of Digital Healthcare, Yonsei University Mirae Campus, 1 Yeonsedae-gil, 26493, Gangwon-do, Republic of Korea

*Corresponding author: youngcho@yonsei.ac.kr

**Supplementary 1. Sensitivity Analysis of τ and λ**

Table S1. Sensitivity analysis of λ and τ across the three datasets.

|  |  | DAVIS | | BioSNAP | | DrugBank | |
| --- | --- | --- | --- | --- | --- | --- | --- |
|  |  | AUROC | AUPRC | AUROC | AUPRC | AUROC | AUPRC |
| $\lambda$ | ${10}^{-3}$ | 0.9214 | 0.6926 | 0.9185 | 0.9211 | 0.8852 | 0.8854 |
|  | ${10}^{-4}$ | **0.9434** | **0.7611** | **0.9292** | **0.9305** | **0.9131** | **0.9149** |
|  | ${10}^{-5}$ | 0.9403 | 0.755 | 0.9188 | 0.919 | 0.9049 | 0.9032 |
| $\tau$ | 0.05 | 0.9413 | 0.7416 | 0.9196 | 0.9236 | 0.9101 | 0.9119 |
|  | 0.1 | **0.9434** | **0.7661** | **0.9292** | **0.9305** | **0.9131** | **0.9149** |
|  | 0.2 | 0.8766 | 0.6018 | 0.9198 | 0.9239 | 0.9046 | 0.9024 |

To examine how the contrastive alignment hyperparameters affect model performance, we conducted a controlled sensitivity analysis over τ ∈ {0.05, 0.1, 0.2} and λ ∈ {10⁻³, 10⁻⁴, 10⁻⁵} across all three datasets. All experiments were performed under the same training configuration used in the main results.

The results in Table S1 show distinct sensitivity characteristics for the two hyperparameters. For λ, the model maintains consistently strong performance for 10⁻⁵ and 10⁻⁴, with a noticeable drop only at 10⁻³. In contrast, τ exhibits a optimum around 0.1, while τ = 0.2 leads to clear degradation. Based on these observations, we used τ = 0.1 and λ = 10⁻⁴ in all main experiments. These settings provided the best and most stable performance across the three datasets.

**Supplementary 2. Analysis of Soft-Attention Weights**

To further examine how TriDTI allocates importance to different modalities during fusion, we analyzed the learned soft-attention weights for all modalities (sequence, structure, and relation) in both the drug and protein branches. Heatmaps were generated for all samples in the DAVIS, BioSNAP, and DrugBank test sets (Figures S1–S3). These visualizations summarize how frequently and to what extent each modality contributes to the final fused representation.


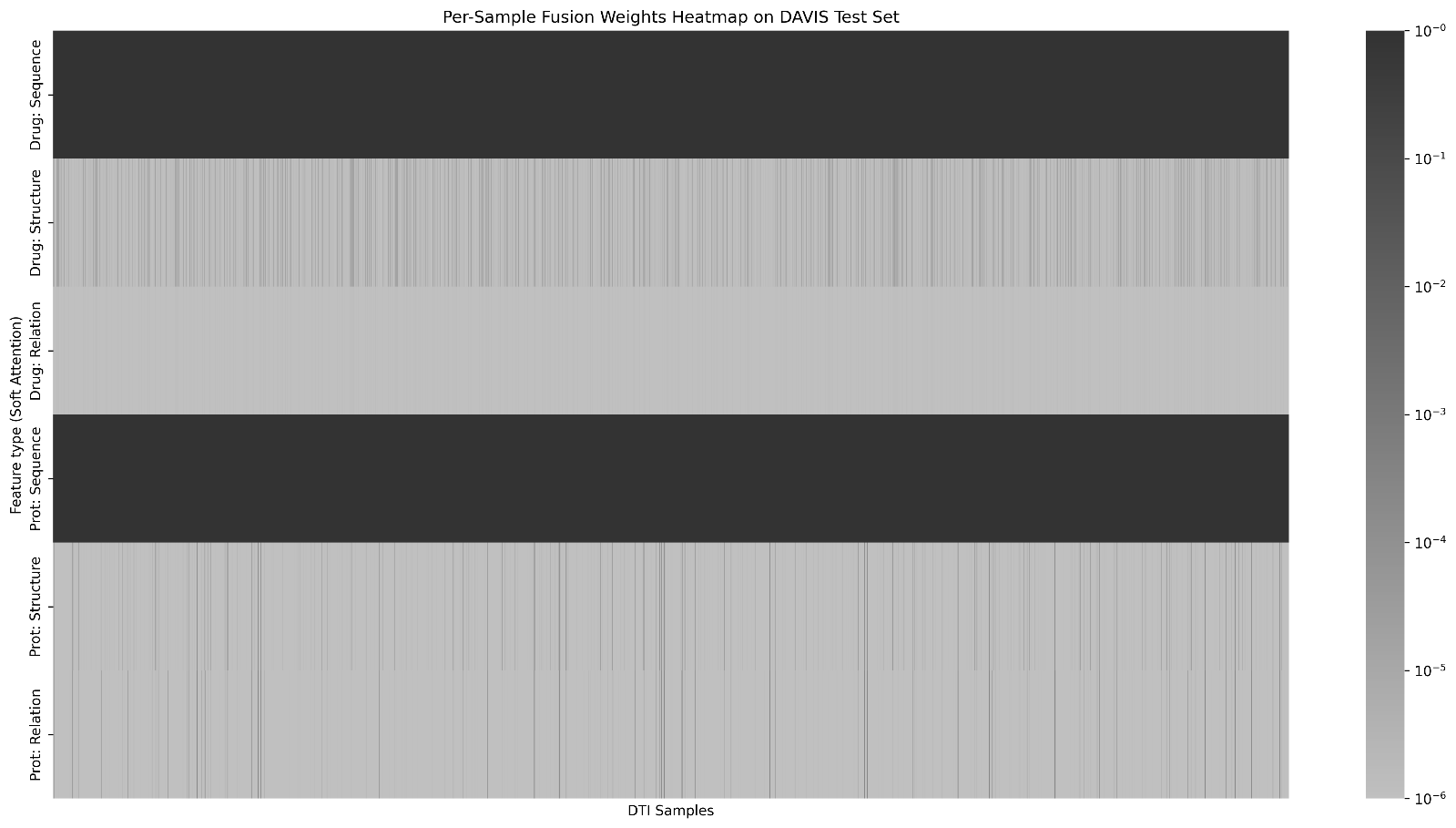


Figure S1. Heatmap of learned soft-attention weights for drug and protein modalities on the DAVIS test set.


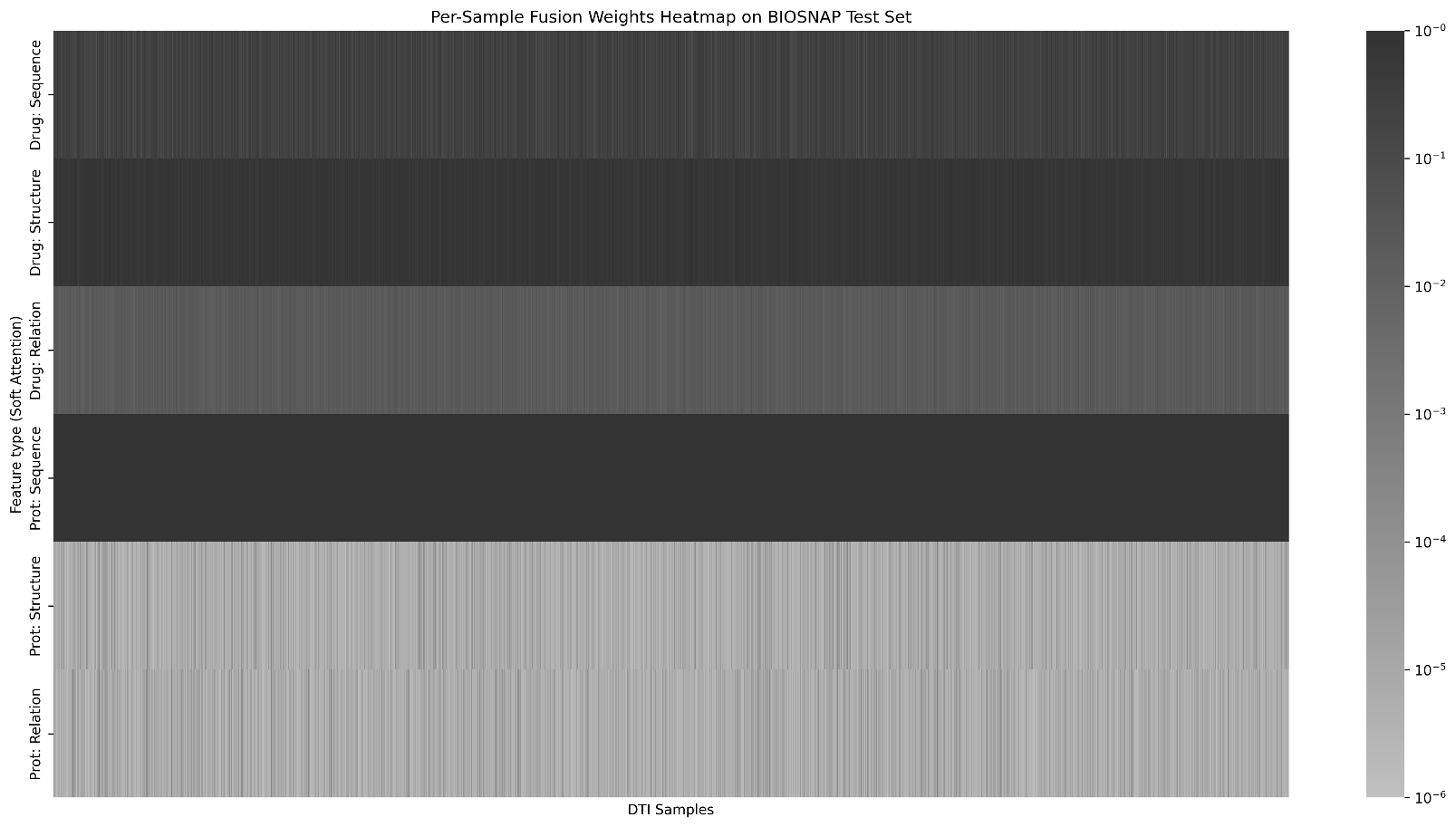


Figure S2. Heatmap of learned soft-attention weights for drug and protein modalities on the BioSNAP test set.


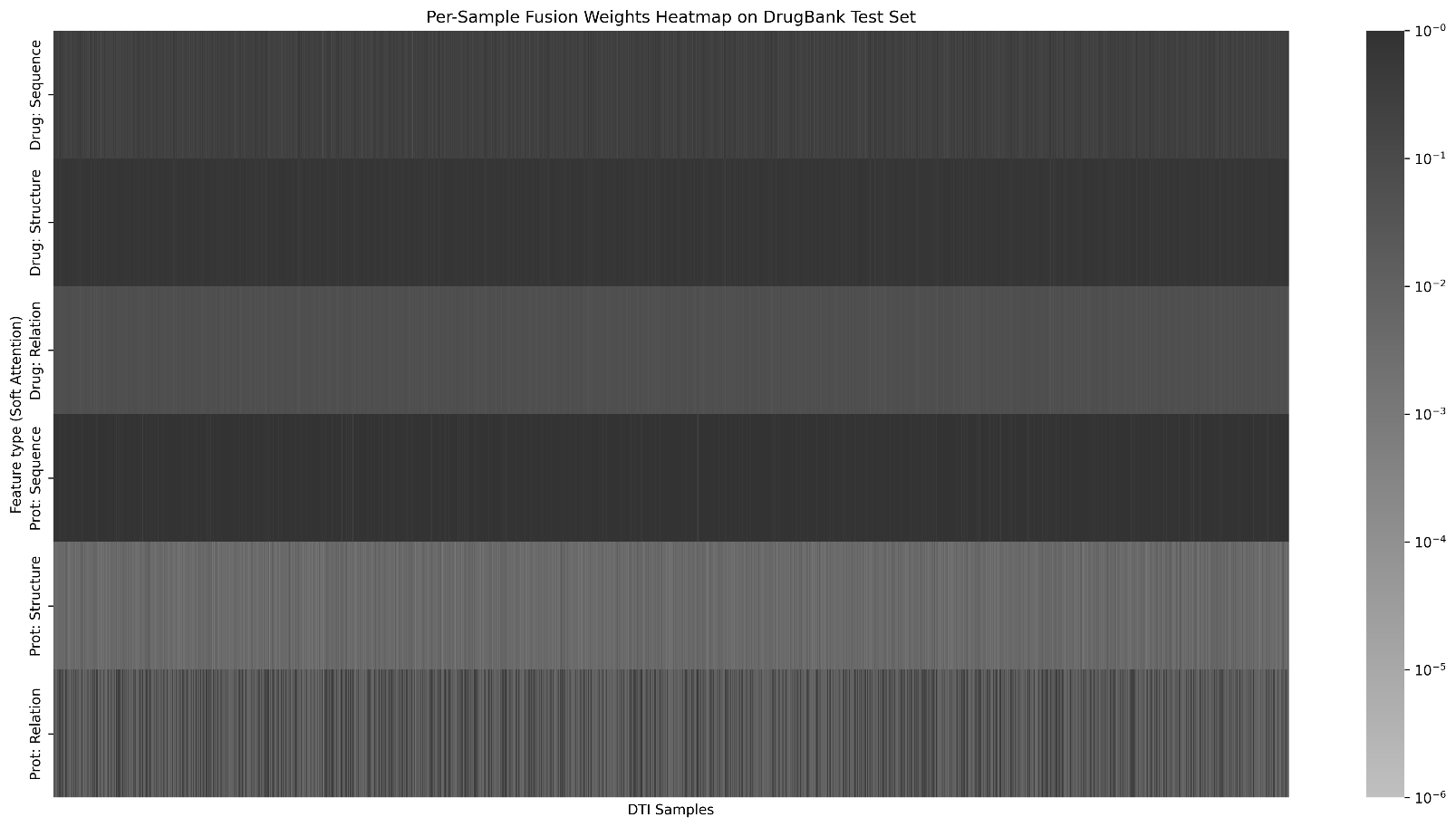


Figure S3. Heatmap of learned soft-attention weights for drug and protein modalities on the DrugBank test set.

As shown in Table 1 on the main manuscript, the DAVIS dataset contains the smallest number of drugs and targets, whereas BioSNAP provides a moderately sized collection, and DrugBank is the largest dataset with the most diverse set of drug and protein entities. The results show that the DAVIS dataset exhibits strong dominance of the sequence modality for both drugs and proteins. This suggests that pre-trained sequence models provide more informative representations when training data are limited, leading the model to assign higher attention weights to these features.

In contrast, for the larger and more heterogeneous DrugBank and BioSNAP datasets, the attention distribution becomes more balanced. Both structural and relational modalities receive higher weights compared to DAVIS, indicating that TriDTI effectively incorporates additional modalities when the dataset provides sufficient complementary information. Especially in DrugBank, relational signals from the PPI network and structural graph features contribute substantially, demonstrating that the model adapts its fusion behavior to the richness of available modality-specific information.

Overall, these results confirm that the sequence modality does not dominate due to a fixed bias but rather due to dataset characteristics. As more informative structural or relational patterns become available, the model allocates attention more evenly across modalities.

**Supplementary 3. Visualization of Drug-Target Cross-Attention Patterns**

To investigate how TriDTI captures interaction-specific relationships between drugs and proteins, we visualized the bi-directional cross-attention weights learned by the model. Specifically, we examined two attention maps: (1) Drug as Query → Protein as Key ($ATTN(D\to T)$), and (2) Protein as Query → Drug as Key ($ATTN(T\to D)$), for the DAVIS and BioSNAP datasets (Figures S4–S5). These heatmaps reflect how strongly each drug attends to each protein, and vice versa.


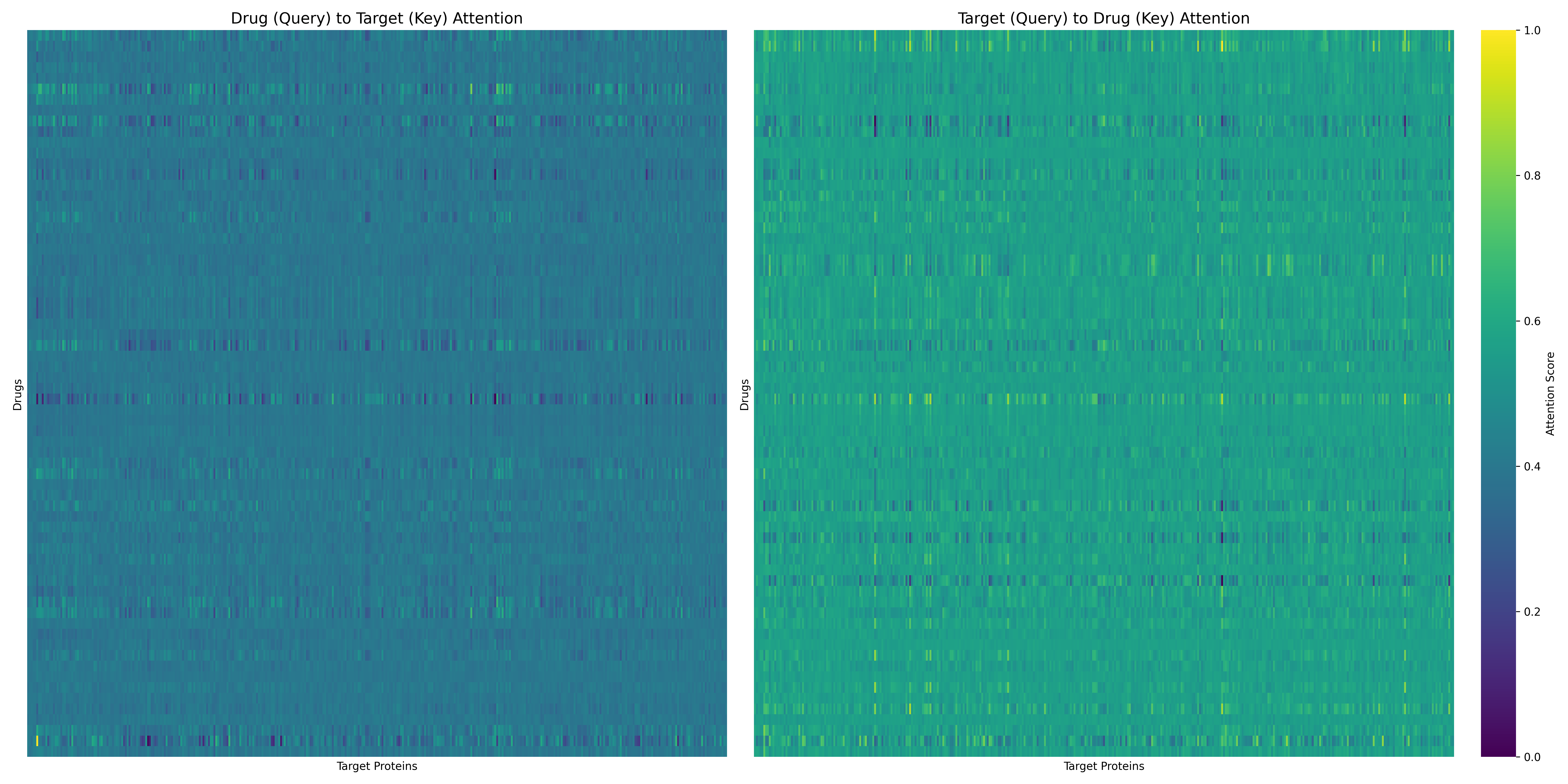


Figure S4. Heatmap of learned cross-attention weights on the DAVIS datasets.


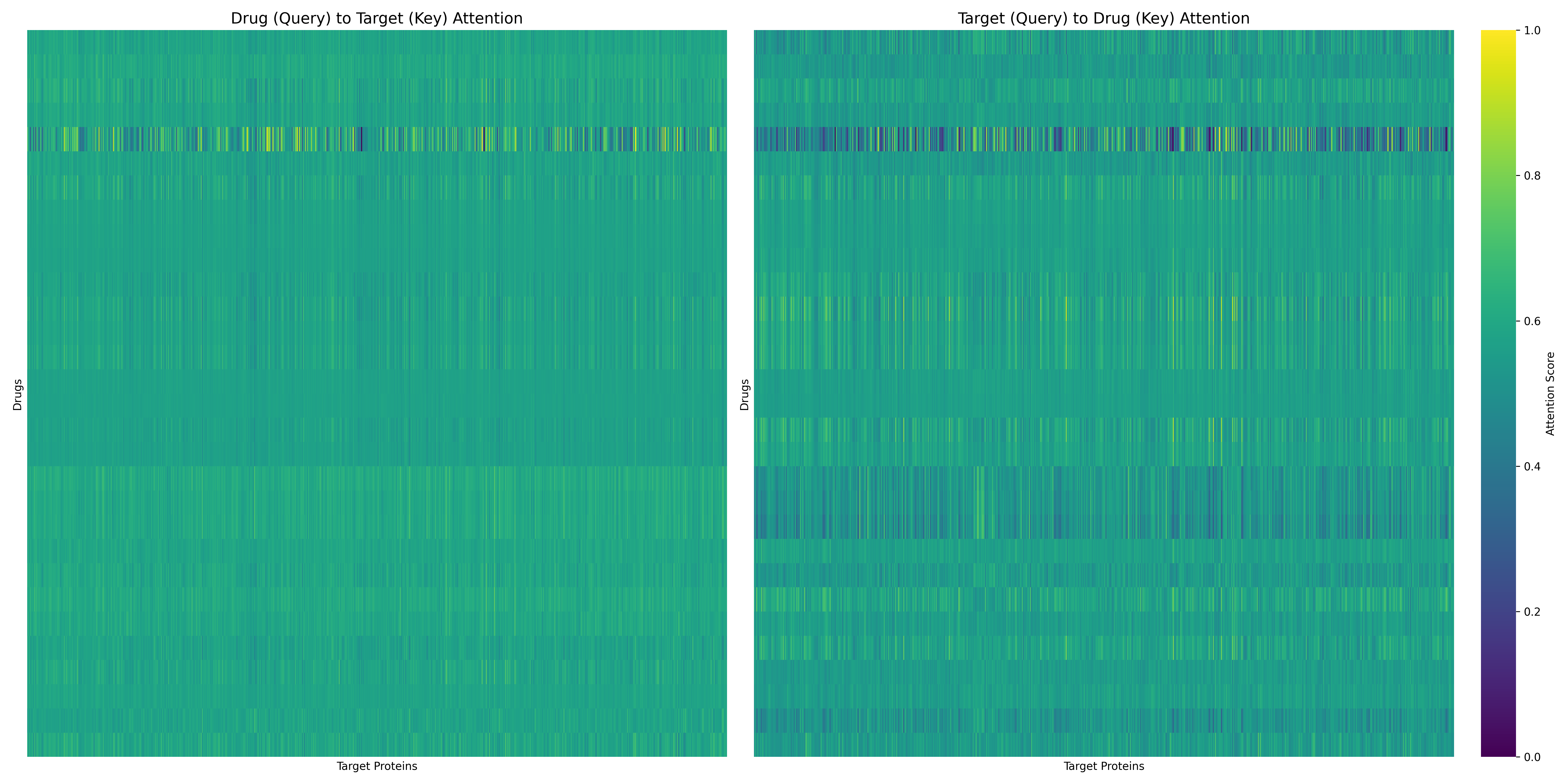


Figure S5. Heatmap of learned cross-attention weights on the BioSNAP dataset.

Across both datasets, the bi-directional attention maps exhibit a clear complementary pattern. Specifically, when the **drug-to-protein attention** ($ATTN(D\to T)$) assigns relatively higher weights to a subset of protein embeddings, the corresponding **protein-to-drug attention** ($ATTN(T\to D)$) tends to assign lower weights to the same drug–protein pairs, and vice versa. This inverse relationship consistently appears across samples and indicates that the two attention directions capture different but complementary aspects of the interaction.

Rather than uniformly attending to the same regions in both directions, TriDTI distributes its attention selectively and asymmetrically, suggesting that each direction emphasizes different informative components of the drug–protein pair. This supports the design choice of using bi-directional cross-attention, as the two directions collectively provide a richer view of potential interaction patterns.

**Supplementary 4. Training and validation convergence analysis**

To further understand the effect of the contrastive learning module on optimization stability and overall training behavior, we analyzed the training dynamics of TriDTI on the BioSNAP dataset over 100 epochs. Figure S6 presents both the training loss curves and the corresponding validation AUROC trajectories for models trained with and without the contrastive objective.


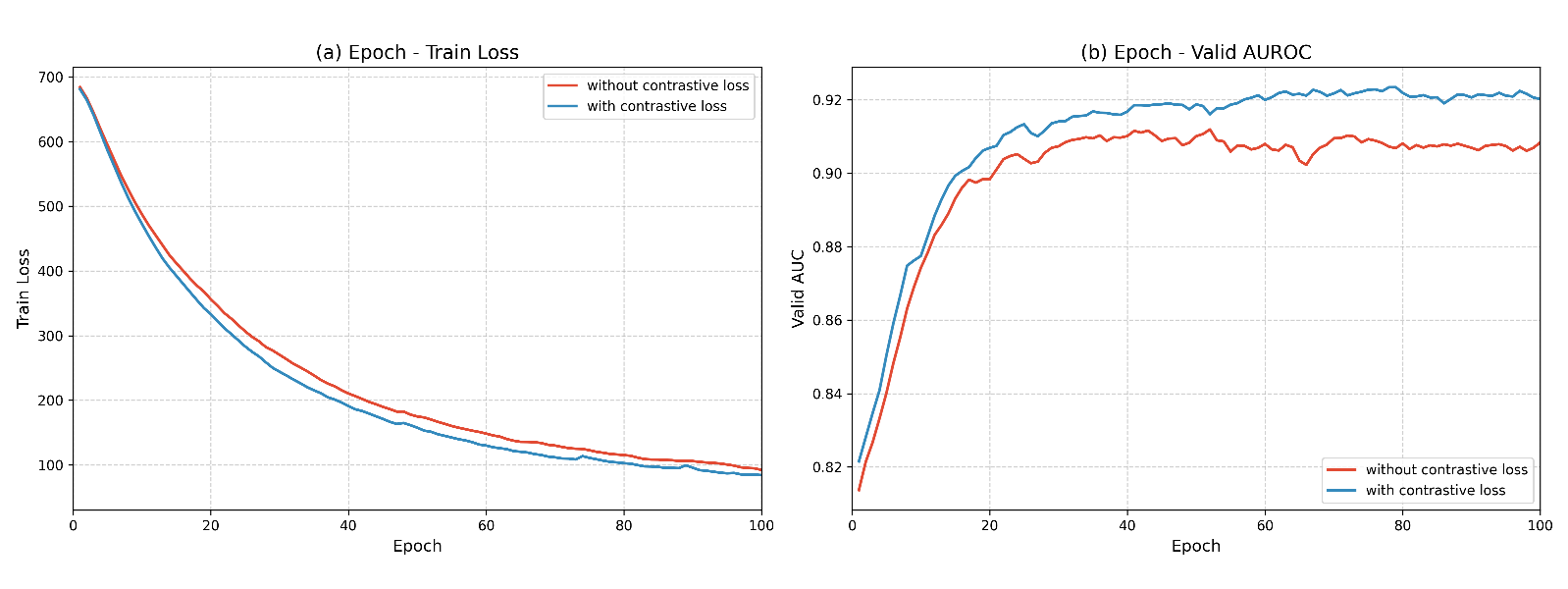


Figure S6. Training dynamics of TriDTI on the BioSNAP dataset up to 100 epochs. (a) Training loss curves for models trained with and without the contrastive learning objective. (b) Validation AUROC curves for the corresponding models.

Clear differences were observed in validation performance. While both models converged, the model trained with contrastive learning consistently achieved higher validation AUROC throughout training and converged to a superior performance level. These results suggest that the contrastive objective contributes to more stable and discriminative representation learning, leading to improved generalization on validation samples.
